# The Roller Bandage

**Published:** 1883-12

**Authors:** 


**The Roller Bandage.** By WILLIAM BARTON HOPKINS, M. D., Demonstrator of Surgery, University of Pennsylvania. With Seventy-three illustrations. Philadelphia: J. B. Lippincott & Co. 1883.

This is a little book of ninety-five pages. It gives a series of definitions and general rules for bandaging, and then proceeds to show how the various bandages are to be applied. Most of the books give elaborate directions for the application of bandages which are usually mystifying; but the author of this valuable little work has, by means of the excellent illustrations, presented the subject so clearly that the student can easily familiarize himself with the details, and with a little practice be able to apply any roller bandage in a workmanlike manner. We would heartily commend this book as the best on this special subject.

